# Person-centred care of people living with dementia and its regulation in German-speaking nursing homes: A qualitative focus group study

**DOI:** 10.1016/j.ijnsa.2025.100400

**Published:** 2025-08-05

**Authors:** Anna Louisa Hoffmann-Hoffrichter, Andreas Hohmann, Bernhard Holle, Rebecca Palm, Martina Roes

**Affiliations:** a German Center for Neurodegenerative Diseases (DZNE), Stockumer Str. 12, 58453 Witten, Germany; b Witten/ Herdecke University (UW/H), Faculty of Health, Department of Nursing Science, Alfred-Herrhausen-Straße 50, 58448 Witten, Germany; c Carl von Ossietzky Universität Oldenburg, School VI – School of Medicine and Health Sciences, Ammerländer Heerstraße 114-118, 26129 Oldenburg, Germany

**Keywords:** Person-centred care, Internal regulations, Nursing home, Long-term care, Mindset development, Dementia, Focus groups

## Abstract

**Background & aim:**

Although nursing homes are aware of the relevance of person-centred care, the translation of this approach into practice remains difficult. This study aims to explore the meaning of person-centred dementia care and the role of internal regulations in German-speaking nursing homes in translating this concept into practice.

**Methods:**

This study (a qualitative exploratory design) is part of an overall study of internal regulations about person-centred dementia care in nursing homes. In this substudy, we conducted nine virtual focus group discussions with experts from theory, practice, and regulatory authorities from Germany, Austria and Switzerland about person-centred dementia care. We analysed the data via qualitative content analysis using a deductive-inductive approach.

**Results:**

For experts, the *leadership function* is crucial for the concept of person-centred dementia care in nursing homes. Leadership is inextricably linked to other elements, such as setting priorities, mindset, structural requirements, internal regulations and outcomes. Leadership therefore has a hinge function: it enables a limited and controlled rotation of connected elements to illustrate the dimensions of the individual elements from person-centred to non-person-centred. In addition to setting priorities and outcomes, mindset development and structural requirements are particularly relevant for the implementation of person-centred dementia care in nursing homes. The experts described differences between traditional internal regulations and regulations about person-centred dementia care in nursing homes. The development of internal regulations for person-centred dementia care and the content of these regulations are consequences of a circular, dialogical collective understanding process. It leads processually in a bottom-up manner to a common understanding of person-centred dementia care, which is then written down. Experts recommend formulating these regulations as concepts and options analogous to mindset development, which employees can use in the care of the resident while maintaining autonomy.

**Conclusions:**

The study provides 1) insight into how person-centred dementia care and the role of internal regulation are understood in German-speaking nursing homes and 2) a precise description of the development of a mindset and regulations for person-centred dementia care in nursing homes that requires both top-down and bottom-up processes.

**What is already known**

• Various definitions of person-centred care exist that make it difficult to provide one universal definition of this approach in nursing.
• The translation of person-centred care into care practice remains difficult.

Alt-text: Unlabelled box

**What this paper adds**

• This article describes how experts from care practice, science and regulatory authorities understand and define person-centred dementia care in German-speaking nursing homes.
• The study participants described the person-centred mindset as an important element in the person-centred care of people living with dementia and specified how mindset development can work in this context.
• The study participants also described what internal regulations for person-centred dementia care should be provided in nursing homes to ensure that employees implement these regulations.

Alt-text: Unlabelled box

## 1 Background

More than 50 % of residents of nursing homes have one thing in common: they are affected by dementia (Hoffmann et al., 2014). With regard to providing good care for people living with dementia in nursing homes, person-centred care is vital (Fazio et al., 2018). In general, person-centred dementia care defined by Kitwood (1997) is a care philosophy that turns away from malignant social psychology, i.e. undermining the personhood of the person living with dementia. Person-centred care acknowledges personhood despite dementia and focuses on the person, including the individual’s needs and preferences. A key aspect is therefore the recognition and perpetuation of personhood in addition to building, fostering, and maintaining relationships (Fazio et al., 2018; Kitwood, 1997; Serbser-Koal et al., 2024). Several authors have examined and further developed the person-centred care approach, including e.g., Nolan et al. (2006) or McCormack et al. (2021), although their focus has not been specifically on people living with dementia. Brooker and Latham (2015) summarize person-centred dementia care in a V,I,*P* + *S* formula: valuing people living with dementia, individualized care, personal perspective, and social environment. The person-centred care approach includes realignment of the institution in the context of a culture change that aims to move away from the institutional biomedical model of care facilities towards a homelike design and, in particular, individualized care to foster high-quality care in nursing homes (Jones, 2011; Koren, 2010).

With respect to the effectiveness of person-centred dementia care compared with usual care, reviews have explored the effects of person-centred care interventions such as e.g., Dementia Care Mapping, staff training, environment change, and music therapy (Chenoweth et al., 2019; Kim and Park, 2017; Li and Porock, 2014; van der Steen et al., 2025). These reviews reveal a reduction in behavioural and psychological symptoms of dementia, such as depression, neuropsychiatric symptoms, and agitation (Kim and Park, 2017; Lee et al., 2022), which also results in an improvement in quality of life (Chenoweth et al., 2019; Kim and Park, 2017).

As awareness of the person-centred care approach has increased, different frameworks to operationalize person-centredness have emerged, such as the Senses Framework (Nolan et al., 2006), the VIPS framework (Brooker, 2007, Brooker and Latham, 2015), and the Person-centred Practice Framework (McCormack et al., 2021). The scientific discourse also shows various definitions of person-centred care that make it difficult to provide one universal definition of this approach in nursing (Byrne et al., 2020). As a result, this concept is often difficult to translate and operationalize into care practice or at the level of health care service (Byrne et al., 2020). This may be a challenge if nursing homes are required and want to implement guidelines (NICE, 2018) or national standards (Deutsches Netzwerk für Qualitätsentwicklung in der Pflege (The German Network for Quality Development in Nursing [DNQP], 2019) for person-centredness in the context of quality of care.

Health care professionals, as well as policy makers, regulators, and administrators, play a significant role in the quality of care. Regulators oversee and control the quality of care in addition to reviewing internal standards (Pot et al., 2024). Therefore, with regard to “compliance regulation” (Pot, 2022, pp. 13–17), nursing homes in Germany establish internal regulations at the structural level to comply with the quality of care. In Germany, several parties monitor the quality of nursing homes: 1) medical service (Medizinischer Dienst) for statutory long-term care insurance or inspection service (Careproof - Prüfdienst der privaten Pflegeversicherung) for private long-term care insurance (Bundesministerium für Gesundheit, 2025). 2) The Housing and Participation Act (WTG-Behörde) authority inspects nursing homes at the municipal and state levels to ensure that they comply with the Housing and Participation Act at the state level (Studier, 2025).

Since there is no general uniform definition of regulations, they can be understood as structured processes (Windholz, 2018). In this study, we use internal regulations as an umbrella term for internal policies, guidelines, and other written internal procedures of nursing homes.

To summarize, it is unclear how German-speaking nursing homes understand and implement person-centred dementia care. Simultaneously, it is unclear how German-speaking nursing homes develop internal regulations of person-centred dementia care and which requirements these kinds of regulations must fulfil to foster the implementation of person-centred dementia care in nursing homes (Hoffmann-Hoffrichter et al., 2024).

### 1.1 Aim

This study explores the perspective of person-centred care experts with regard to the meaning of person-centred dementia care and internal regulation in German-speaking nursing homes in relation to the following questions: Which aspects of person-centred dementia care are of particular relevance in German-speaking nursing homes? What are the internal regulations for person-centred dementia care in German-speaking nursing homes?

## 2 Methods

### 2.1 Study design

We designed this substudy as a qualitative exploratory study, which is embedded in an overall study of internal regulations about person-centred dementia care in nursing homes. In the overall study, we 1) explored the meaning of person-centred dementia care and 2) examined the content validity of the Dementia Policy Questionnaire, which aims to measure the existence of internal regulations about person-centred dementia care. The Dementia Policy Questionnaire has been described in detail elsewhere (Hoffmann et al., 2022, Hoffmann-Hoffrichter et al., 2024). In this substudy, we conducted virtual focus group discussions with experts in person-centred care from Germany, Austria and Switzerland to explore the meaning of person-centred dementia care. We wanted to capture and understand heterogeneous perspectives in an interactive and dynamic discussion environment and explore the topic in depth in groups. Focus group discussions provide a social space in which shared experiences based on existential commonalities are articulated, represented, debated and changed by group interactions (Barbour, 2018; Jayasekara, 2012; Kühn and Koschel, 2011). The researcher predetermines a topic, and the data are obtained through the interactions of the participants (Kühn and Koschel, 2011; Lamnek, 2005). The underlying theoretical approach of focus groups is the interpretative paradigm, where new meaning is generated on the basis of the participants’ perspectives and interactions during focus group discussions (Jayasekara, 2012).

We chose the data collection method of virtual focus group discussions, where participants meet online via a video conferencing tool (Tuttas, 2015), since the potential participants in this study were located in various geographic regions. For the reporting of this study, we followed the consolidated criteria for reporting qualitative research (COREQ) checklist for interviews and focus groups (Tong et al., 2007).

### 2.2 Participant selection

#### 2.2.1 Sampling

We recruited participants from July 2023 to January 2024 using a purposive sampling technique (Polit and Beck, 2017) to identify experts on person-centred dementia care. We included experts with practical experience, theoretical experience, and experience in evaluation who met the following inclusion criteria: 1) scientific experts on person-centred dementia care were included if they had conducted conceptual and/or empirical research on the topic; 2) practice experts on person-centred dementia care were included if they were in a senior or middle management position or if they were care professionals in a nursing home according to SGB XI §72 and had provided dementia care for a minimum of six months; and 3) experts on regulatory authorities were included if they worked in a quality care commission authority and assessed or inspected nursing homes according to SGB XI §114 and the Housing and Participation Act for every federal state. As we were able to identify only a few scientific experts in Germany, we also recruited scientific experts from Austria and Switzerland for one focus group. All the experts had access to computers with stable internet connections. To avoid hierarchies and speaking blocks, we divided the scientific experts, practice experts, and experts on regulatory authorities into three subgroups.

We recruited all participants from our network and conducted an intensive internet search in addition to the snowball technique. To recruit practice experts, we used a list of all nursing homes in Germany (Pflegemarkt.com, 2021).

#### 2.2.2 Method of approach

We first contacted 114 persons via mail with a personal cover letter, a study information document containing reasons for conducting the research, and an informed consent document. We contacted potential participants by telephone, if possible, to inquire about their interest in participating in the study. The contacted persons had the opportunity to ask questions about the study at any time. All participants provided written informed consent.

The reasons for nonparticipation among scientific and practice experts were a lack of availability, time and personnel resources, and sickness-related absences. Employees from regulatory quality care commissions were either unavailable or stated a lack of interest or time resources when they declined participation.

#### 2.2.3 Sample size

Following the recommendations of Krueger and Casey (2014) and Kühn and Koschel (2011), we planned to conduct two to four focus groups per subgroup as ad hoc groups, which are put together specifically for focus groups and enable a dynamic discussion and the presentation of opinions (Kühn and Koschel, 2011). To encourage discussions of different perspectives and experiences (Barbour, 2018), we sought heterogeneity within these subgroups with regard to sociodemographic criteria such as age, professional experience and the location of employment.

For virtual focus group discussions, a smaller sample size is recommended compared to face-to-face groups (Mann and Stewart, 2000; Strout et al., 2017). For each subgroup, we planned to include approximately five to seven participants. In accordance with Lambert and Loiselle (2008) and Taylor (2005), we offered the participants individual interviews if they were unable to participate during the defined time frame.

#### 2.2.4 Setting

We conducted virtual focus group discussions and virtual individual interviews with the Zoom© videoconferencing platform and provided the participants with a link to attend the discussion.

#### 2.2.5 Data collection

We collected data via focus groups. In exceptional cases where participants were unable to participate in the focus groups, we conducted virtual individual interviews.

The first author contacted the participants by mail to discuss a convenient time for the focus group discussions and made two appointments with each focus group: 1) a first appointment to discuss the meaning of person-centred dementia care in nursing homes and internal regulations and 2) a second appointment for the focus group-based cognitive interviews to supplement the Dementia Policy Questionnaire (Hoffmann et al., 2022). The results of this study focus exclusively on the first appointment.

Two weeks before the arranged focus group discussions, the study participants received a fifteen-minute online questionnaire via LimeSurvey (LimeSurvey, 2024) (Appendix S2). The questionnaire included sociodemographic and general questions on the research topic to ask about the participants’ understanding of internal regulations.

For the virtual focus group discussions, we developed a semistructured interview guide that targeted the specific subgroups on the basis of the findings of our previous studies (Hoffmann-Hoffrichter et al., 2024; Hoffmann et al., 2022). We pretested the interview guide with the first focus group with respect to clarity, wording of the questions and timing and made small adjustments. We included the pretest in the data analysis.

During the virtual focus group discussions, we used the interview guide for orientation. For the virtual individual interviews, we did not create a separate interview guide but used the topics from the focus group discussion interview guide. We planned the virtual focus group discussions to be approximately 90 to 120 min long. In the Appendix (S3), we provide the semistructured interview guides that we translated verbatim from German.

We audio recorded all the virtual data collection using a separate audio recorder and automatically transcribed the audio recordings via NoScribe software version 0.4.1 (Dröge, 2024). The first author compared every audio file with the automated transcript. We stored the data in the protected file server environment of the German Center for Neurodegenerative Diseases (DZNE), to which only the persons involved in the project had access.

### 2.3 Data analysis

We coded the transcripts via MAXQDA 22.0.1 software (VERBI Software, 2021). The first author and the second author analysed the transcripts by applying a qualitative content analysis method using a deductive-inductive approach (Kuckartz and Rädiker, 2022).

To become familiar with the data, we independently read the transcripts thoroughly. The first author used the field notes from the focus group discussions to better understand the context. The first author wrote short case summaries and memos during the analysis. In accordance with the questions of the interview guidelines, the first author deductively developed themes and generated a main category system on the basis of the transcripts of three interviews. Initially, the main categories derived from the interview guide included 1) individual meaning of person-centred care; 2) meaning of person-centred care for nursing homes; 3) dementia-specific versus traditional care; and 4) internal regulations on person-centred care in nursing homes. The first author generated code definitions and added text passages as examples. To improve the reliability of the coding, the first author and the second author validated the main category system by coding three interviews independently. On the basis of the discussed results, the first author adapted and specified code definitions and coded all transcripts with the main categories. To differentiate the main categories, the first author inductively developed subcategories and enhanced and specified the category system with the subcategories, their definitions and examples on the basis of the material. The first author validated and discussed the codes with the entire study team. To maintain quality and reliability, the first author and the second author concurrently coded the material in the enhanced category system on the basis of transcripts of seven interviews. From April to June 2024, the first author and the second author met on a weekly basis and discussed all of the coding. In cases of discrepancies, discussion was conducted until a consensus was reached. If a consensus could not be reached, the research team (Author 3, 4, 5) was consulted to reach a consensus. The first author subsequently coded all of the material. Since the dataset of the virtual individual interviews was not equivalent to the dataset of the focus group discussions, the final step involved coding the transcripts of the virtual individual interviews according to the same category system and comparing them with the results of the focus group discussions for congruence.

To ensure intersubject comprehensibility, we discussed the entire research process and the results of the data analysis in monthly meetings with the research team. To adopt the peer debriefing approach, the first author reflected on the project at regular intervals with colleagues from the German Center for Neurodegenerative Diseases (DZNE), who were not involved in this project. For more information about the quality criteria, see Appendix S10.

## 3 Ethical considerations

Ethical clearance for this study was obtained from the German Society of Nursing Science in March 2023 (application number: 23–002).

## 4 Results

The sample included 36 participants. We conducted nine virtual focus groups with 34 participants and two virtual individual interviews with two participants. We present their demographic characteristics in Table 1. We composed four virtual focus groups for experts on theory, three virtual focus groups for experts on practice, and two virtual focus groups for experts on quality care commission. The analysis of the two virtual individual interviews was congruent with the results of the focus group discussions, and no additional codes emerged. The duration of the focus group discussions ranged from 76 min to 114 min (mean 92 min), whereas the duration of the virtual individual interviews ranged from 68 to 145 min (mean 106.5 min).

**Table 1:** Demographic characteristics of the focus group discussion participants (*n* = 34).

Demographic characteristics	FGD 1	FGD 2	FGD 3	FGD 4	FGD 5	FGD 6	FGD 7	FGD 8	FGD 9	VII*
**Country**	D	D	A-CH	D	D	D	D	D	D	D
**Subgroup**	SE	SE	SE	SE	PE	PE	PE	RA	RA	SE
**Number of Participants**	4	6	5	2	4	3	3	4	3	2
**Age in years mean (range)**	42.75 (35–60)	50.17 (39–61)	50.4 (33–63)	56.0 (50–62)	50.25 (37–58)	43.33 (40–46)	37 (24–44)	55 (44–62)	46.67 (42–55)	62 (60–64)
**Gender**										
Male	3	2	1	1	2	2	1	3	1	2
Female	1	4	4	1	2	1	2	1	2	0
**Job function**										
Graduate working in research	3	3	3	2						1
Graduate working in education, consulting, innovation	1	3	2							1
Staff in nursing home management					4	2	2			
Registered nurse in nursing home						1	1			
Staff in quality care commission management								2	1	
Employee quality care commission								2	2	

Legend.

FGD = Focus group discussion.

VII = Virtual individual interviews.

*D* = Germany.

*A* = Austria.

CH = Switzerland.

SE= Scientific experts.

PE= Practice experts.

RA= Regulatory authorities.

⁎ Two persons who were originally assigned to focus group 4 were unable to take part in the focus group discussion.

**Fig. 1:**
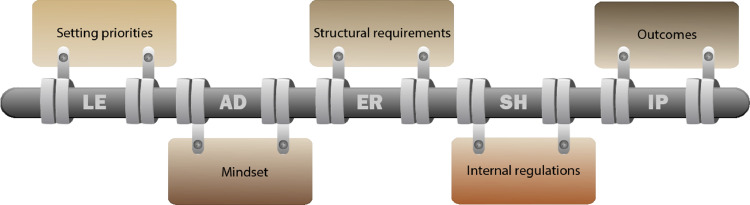
Overview of the six main categories.

With respect to the first research question, the experts identified several aspects of person-centred dementia care that are particularly relevant in German-speaking nursing homes. During the data analysis of all nine virtual focus group discussions, we identified these aspects of person-centred care as six main categories: leadership, setting priorities, mindset, structural requirements, internal regulations, and outcomes (Fig. 1). With respect to the second research question, the internal regulations category describes regulations for person-centred dementia care in German-speaking nursing homes. The experts discussed the content of the categories at different intensities; therefore, the reporting for each category varied. Appendix S4 shows the category system with exemplary quotes.

### 4.1 Leadership − the hinge of person-centred care

For experts, the *leadership function* is crucial for the concept of person-centred dementia care in nursing homes. Leadership is inseparably linked to the elements of setting priorities, mindset, structural requirements, internal regulations, and outcomes. On the basis of the data, leadership is best described as a hinge function that can be either a requirement or an influencing or reactive factor for person-centred care. As a hinge, leadership enables a limited but controlled rotation of connected elements. The hinge itself illustrates the facets as rotations of the individual elements.

#### 4.1.1 Management levels

The experts described staff members at different management levels who were perceived as leaders and provided leadership. This included people at the top management level, such as managing directors and nursing home directors, as well as people at the middle level of management, such as nursing home managers and area managers. At the lower management level, care unit managers have a significant influence on the team with regard to the implementation of person-centredness.

#### 4.1.2 Leadership qualities

The experts explained that, to rotate the connecting elements in a person-centred direction with leadership as a hinge, various leadership qualities based on a person-centred mindset are needed. These include dedication; person-centred employee management, including facilitative leadership and role model functions; and the continuity of the leader as well as leadership skills (Appendix S5).

*“Above all, you need a very committed manager that follows up on this, that drives it forwards and supports it to a certain extent. […] Because in my experience […] the fish rots from the head. If it doesn't work, it's often because the management doesn't follow it up.”* (Focus-Group-1-Expert00)

#### 4.1.3 Leadership consequences

From the experts’ perspective, leadership influences the degree to which person-centredness is implemented in a nursing home. Additionally, managers can build and maintain relationships with employees through an appreciative approach. This ensures staff continuity and helps ensure that role models who unofficially exemplify person-centredness remain at the nursing home.

*“[…] that the providers set themselves up conceptually in such a qualitative way that they can ultimately create a relationship with the employees […], and thus the spiral goes upwards, as it were, because the fact that the quality on site is oriented upwards means that employee satisfaction is greater […].”* (Focus-Group09-Expert01)

Further consequences of these leadership qualities become evident in the other elements of person-centredness.

### 4.2 Setting priorities - ranging from embedding to decoupling person-centred care in a nursing home

For the experts, setting priorities represents a nursing home’s decision for or against a person-centred approach at the organizational level. This decision is made by people who have a leadership function.

#### 4.2.1 Prioritization of person-centredness

According to the experts, nursing homes weigh their position for or against person-centred care on the basis of external factors and factors at an organizational level, such as the requirements of management. Whether facilities, including management and support for employees, prioritize person-centred care depends on their requirement to be person-centred. The prioritization of person-centredness can manifest in the implementation of person-centred structural quality, but this does not guarantee the actual implementation of this approach.

*“I would say it depends on the provider and what they want. That's where it starts to matter whether person-centred care is interesting in any way.”* (Focus-Group-8-Expert03)

#### 4.2.2 Lacking prioritization of person-centredness

While smaller nursing homes that do not belong to a large provider can usually act more autonomously, nursing homes run by large, nationwide, profit-oriented providers are other-directed if their executive management has already carried out prioritization. By arguing that person-centred care is too expensive and that regulatory authorities do not examine it, provider and nursing home directors prioritize the profit orientation of a company over providing care that is focused on the resident as a person. The contradiction becomes concrete, as nursing home managers, even if they prioritize person-centred care, cannot implement person-centredness due to a structure that is driven by the managing director. Nursing homes do not prioritize person-centred care if they attach more importance to the ratings of regulatory authorities than to the ratings of residents.

*“It culminated in the sentence that my NHM [nursing home manager] said to me: if I have to decide between the requirements of the MRB [medical review board] and those of the residents, I will always choose the MRB. […] [T]here is often such a fear-driven attitude, especially at the level of those responsible, […].”* (Focus-Group-8-Expert01)

### 4.3 Mindset – ranging from development to stagnation

For experts, mindset includes the behavioural practices of the staff with the aim of developing a person-centred culture in the nursing home.

From a theoretical frame, person-centred mindset development aims to achieve a common person-centred mindset in the nursing home. Due to influencing factors, the collective understanding process and mindset work results in a never-ending, mutually dependent cycle:

*“Actually, what the concept is has to be constantly re-established.”* (Focus-Group-2-Expert00)

#### 4.3.1 Influencing factors

From the experts’ perspective, influencing factors at the societal level are awareness and recognition of people living with dementia and care for people living with dementia. Influencing factors at the personnel level are the generation of employees and their expertise through training and the expert standard as well as their practical experience. At the individual level, the personal mindset of each employee is an influencing factor.

*“You always have some kind of understanding or some kind of mindset. However, if it runs contrary to the considerations of person-centred care and I come in with the mindset, so actually it's only about being full and clean […] then it wouldn't be possible.”* (Focus-Group-4-Expert02)

#### 4.3.2 Collective understanding process

The collective understanding process includes all efforts to develop a common understanding of person-centred care and to find a common translation of person-centredness for the nursing home. For experts, mindset development may stagnate if the leader does not initiate and support this process with the staff. Leadership promotes collective understanding processes by acting as a role model to initiate and implement person-centred care through communication, by regularly exchanging thoughts and ideas with employees, by reflecting on their personal understanding of person-centred care and person-centred leadership, and by promoting shared dynamics as facilitators by empowering employees. The collective understanding process is process orientated and requires continuous updating and maintenance due to the influencing factors. To achieve a successful collective understanding process in a nursing home, three key factors are relevant: including all the perspectives, communicating across hierarchies, and sharing dynamics (see Appendix S6).

*„I would focus not only on the residents, but also on communication in the entire facility. I would understand this to include communication among the employees, communication from the top management to the base, as well as the other way around, as well as considering the residents and relatives. Therefore, the overall topic of communication, how does this take place?“* (Focus-Group-9-Expert01)

### 4.4 Mindset work

Collective mindset development requires collective mindset work, which refers to the mindset and implementation of a shared understanding that emerges in action and interaction. The experts described detractions that revealed stagnation in mindset development. At the same time, they described forms of person-centred mindset work. Appendix S7 shows the different aspects of mindset work, which include designing the environment, understanding the resident with dementia as a person, individualizing care, changing perspectives, providing activity supplies, and designing relationships.

*“For me, this aspect of initiating a relationship is really something very essential, important, that I, do it really well… I really think about it for each resident, discuss it in the team, even if someone has found access […] that it doesn't, remain his secret, […].”* (Focus-Group-5-Expert-04)

Managers promote mindset work by treating employees with respect, acting as role models for mindset work and monitoring the implementation of a collective mindset.

#### 4.4.1 Preliminary consequences of collective mindset development

For experts, the preliminary consequences of the collective understanding process and mindset work are assigned to the organizational collective as well as the individual level. These consequences are preliminary, as the team produces them constantly and repeatedly in a loop. At the organizational, collective level, one consequence is a shared understanding, which forms a basis for further operationalizing person-centred care. A shared understanding of normality can differ between a dementia care unit and usual care units within a nursing home. At the same time, a shared understanding provides the basis for the integral awareness of the staff, which allows person-centredness to become meaningful for the nursing home and allows a person-centred mindset to emerge that is continuously expressed in the actions of the staff. This can lead to the culture development in the nursing home, in which a common understanding is repeatedly established and adopted by everyone. A further consequence appears in the design and dimensions of internal regulations.

*„Therefore, for me, something like an understanding is something that actually has to be established again and again every day through certain process specifications.”* (Focus-Group-2-Expert00)

### 4.5 Structural requirements − from self-determination to heteronomy

Structural requirements involve all aspects of structural quality in the nursing home that are relevant for person-centred dementia care. According to the experts, these aspects have a positive or negative influence on the implementation of person-centred dementia care in nursing homes. If structural requirements have a positive influence on implementation, employees can act in a self-determined way.

#### 4.5.1 External structural requirements

From the perspective of the experts, external structural requirements influence the internal structure and regulation of nursing homes. These include the claims and demands of relatives, external regulations that involve structural quality criteria whose implementation regulatory authorities require and assess, and national expert standards. Each facility reacts individually to external structural requirements due to its individual ecosystem, including management decisions as well as history, architecture, staff, residents and the environment. The implementation of structural quality criteria generates less of a mindset change than pressure in nursing homes, with which the regulatory authorities produce their own mindset of care. The regulatory authorities perceive mandatory audits as snapshots that do not really present realistic conclusions about the actual care provided in the nursing home.

*“We often experience this in our day-to-day work, where everything is made to look pretty for us, for the exam or the documentary or whatever, but the mindset and awareness isn't really there.”* (Focus-Group-9-Expert01)

#### 4.5.2 Institutional structure

According to the study participants, structural requirements also include the institutional structure, which comprises the structure of the facility, personnel and care. We describe this in detail in Appendix S8. Leadership can have a positive influence on the facility structure by reacting to changing framework conditions, reflecting on them and committing to implementing person-centredness while facilitating training opportunities and taking employees on board with practical support. Leadership can have a positive influence on personnel structure by reflecting on personnel resources, separating from employees who do not have a person-centred mindset and avoiding fluctuations in staffing. Furthermore, leadership can positively influence and direct care concepts by promoting person-centredness with creative will.

*“A resident living with dementia […] is no longer mobile, and that then has to be removed from the care unit, because the insurance company has said that this increased need for care, […] is then no longer given for the bedridden resident […]. […] I've always been able to keep my hand over it and haven't had to move anyone when they're in palliative care or even in the final phase. […]. So that's another one of my heart's desires that I have for myself and that I have to or would like to implement in this care unit.”* (Focus-Group-6-Expert02)

### 4.6 Internal regulations – range from micro standards to a person-centred toolbox

The experts described various dimensions of internal regulations, such as definitions, relevance, and characteristics, as well as regulations for person-centred dementia care.

#### 4.6.1 Types of regulations

Different types of conventional regulations differ in their orientation and concretization. Mission statements are regulations that are both externally and internally oriented with a low degree of concretization and include, for example, management principles or vision. These statements are documents in written or short video form. Regulations that combine internal and external requirements have a higher degree of concretization and can be found in, for example, quality manuals. Regulations that are directed internally only have the highest degree of concretization and are operationalized in written procedures and structures, such as guidelines and instructions. Experts call these microstandards, as they prescribe specific nursing actions that employees must follow. Nursing records are a way of regularly providing information about nursing actions, care provision and the execution of or deviation from care actions.

#### 4.6.2 Relevance of regulations

The experts see the relevance of regulations with respect to various aspects. Regulations are necessary to bring evidence-based knowledge into practice and thus to establish and maintain the professionalization of nursing. Furthermore, regulations are relevant tools for external representation to address the external structural requirements that regulatory authorities request. As a result, regulations provide employees with process safety, especially if there is a risk of losing a common understanding due to staff fluctuations. Under these conditions, employees can practice regulations in the nursing home.

*“I think it is important that there is process security in institutions […]. This means that there needs to be clarity in institutions, that it should be regulated who is in what role when, who has what task when?”* (Focus-Group-1-Expert-01)

#### 4.6.3 Characteristics of good regulations

Experts attribute different characteristics to good regulations that are implemented in practice. Regulations should be available in an updated and digitized form. They should be compact and should have a manageable number of rules and prioritized content. In addition to being practical and easy to use, regulations should be comprehensible for employees. Furthermore, regulations should be specific to a target group. For regulations on person-centred dementia care, experts reject standardization-binding rules, so-called microstandards, which restrict autonomy:

*“If you make a microstandard like this, how do I deal with a person with dementia, it is more or less in the sense of what you just said, to the* max*. This autonomy, both the autonomy of the care professional and the autonomy of the recipient of care, is killed by these standards.”* (Focus-Group-8-Expert04)

#### 4.6.4 Regulations on person-centred dementia care

Some nursing homes create internal conventional regulations in a top-down manner that can lead to feelings of a loss of autonomy for staff and care recipients. Several experts prefer a bottom-up approach for the generation of regulations: In this approach, on the basis of the theoretical foundation of person-centred care, assistants, care professionals, quality management officers and managers jointly develop internal regulations as structured and written documentation of their mindset through a circular, dialogical collective understanding process. This leads to a common understanding of person-centred care.

*“Perhaps an essential element […] is that employees are involved in the development of these […] regulations. That they are not necessarily prescribed top-down, but that we set out together, based on whatever kind of initial understanding of […] person-centred care for people living with dementia, and then work together to develop them.”* (Focus-Group-1-Expert-04)

To implement regulations on person-centred care, they need to be communicated within the nursing home. Staff are allowed to critically question the common understanding documented in the regulations at any time. These reflective processes are stimulated with the help of various tools that serve as dialogue engines, such as reflection circles, ad hoc case conferences and monthly meetings. Regulations are temporary as they are developed in a process-like manner from the institution itself, to which employees are committed. Leadership plays a special role; it is the responsibility of the manager to establish and maintain a balance between quality assurance and basic understanding in the facility.

*“And I think that the management or leaders of such an institution naturally have a special responsibility because they have to make it clear that one thing is the quality discourse, which is mandatory and defined externally, and the other is our basic understanding or our basic mindset.”* (Focus-Group-4-Expert01)

For the content of regulations on person-centred dementia care, the theoretical framework of person-centredness should be anchored in different regulations. Regulations should be formulated as concepts and supplies, including aspects of the collective understanding process and mindset work, while still maintaining autonomous action. The experts also identified concepts and options of mindset work that should reflect the regulations on person-centred care. Regulations that describe aspects of the personnel structure and structural features including aspects of the facility structure, such as process descriptions, and information training opportunities, are also necessary.

The understanding of regulations in person-centred dementia care differs from the understanding of other conventional regulations. For experts, regulations on person-centred dementia care include structured written documentation of their common understanding and mindset. This written documentation reflects the common understanding of normality, for example, in a code of conduct or in house rules:

*“[T]here are various sentences and rules that you ask the visitors for. […], self-determination, well-being, being able to let go, your own world of experience, being accepted.”* (Focus-Group-6-Expert00)

The experts also identified an alternative term for action-oriented regulations. They proposed the term “toolbox”, a written framework that provides concrete suggestions for mindset work in various situations. These tools include options for dealing with people living with dementia or options in the activity area. At the same time, the experts emphasized the need for regulations on person-centred care to initially reflect the multidimensionality of this approach in a generalization that can be specified as the process progresses.

*“On the one hand, to provide a framework, but on the other hand, to give people the freedom to move within this framework and find a good balance. That is probably really just a process that has to be negotiated again and again.”* Focus-Group-2-Expert05

### 4.7 Outcomes from person-centred development

The outcomes include all the results that emerge from a person-centred culture in a nursing home that is influenced or driven by leadership. The experts reported outcomes of person-centred care at the facility level and at the individual level.

*“It would not only have a positive influence […] on the care of people living with dementia, but also on the employees and their self-concept. And so this has many side effects, such as perhaps employees’ satisfaction in their jobs, motivation, staff loyalty. All these things are, so to speak, long-term factors that can then achieve a significance that, in addition to the direct care of people living with dementia in the interaction […] can also develop a significance for such an institution.”* (Focus-Group-4-Expert02)

#### 4.7.1 Outcomes at the facility level

One outcome at the facility level is the atmosphere in a nursing home, which is linked to feelings of familiarity and a culture of closeness and warmth. For staff, the atmosphere is linked to satisfaction and the enjoyment of coming to work. The staff develops self-confidence in their knowledge and expertise and is responsible for the decisions they make. Through the development of their professionalism, they obtain the expertise, flexibility and competence to find an individual approach to the person living with dementia, to respond to them individually and to live a reflective practice as an expression of their person-centred mindset.

*“And professionalism, because I simply believe that this is truly professional care, when we have the ability to respond individually to this situation, to act, this reflective practice in the situations.”* (Focus-Group-3-Expert05)

#### 4.7.2 Outcomes at the individual level

At the individual level, self-efficacy is a consequence of collective mindset development for both the person with dementia and the staff. This can be expressed, for example, in the behaviour of the person living with dementia. For employees, it can be expressed in their ability to achieve a positive effect on their behaviour, such as in the actual implementation of care planning. Person-centred care has also a positive influence on people living with dementia by enabling them to lead a good life, as they understand it, in the nursing home. This means that residents feel accepted in the nursing home, feel that they are part of a community and demonstrate a sense of well-being.

*“And the goal, the whole person-centred care aims to ensure that someone has a good life. […] What the person understands by that is up to them. They have to find out to a certain extent. It's very different. It's individual, but that's what it's all about.”* (Focus-Group-1-Expert00)

## 5 Discussion

The aim of this study was to explore the meaning of person-centred dementia care in German-speaking nursing homes and the role of internal regulation from the perspective of various experts. The results reveal that leadership takes on a hinge function that is directly linked to five elements. In addition to setting priorities and outcomes, the development of a shared mindset and the impact of structural requirements are particularly relevant for person-centred dementia care in nursing homes. Experts also identify the dimensions of internal regulations.

With respect to the role of leadership, specific leadership qualities are central to fulfilling a hinge function through managers’ influence on various elements. Leadership engagement is a relevant factor in implementation frameworks in general (Damschroder et al., 2009) as well as in the implementation of person-centred care interventions, such as Dementia Care Mapping (Quasdorf and Bartholomeyczik, 2019).

The characteristics identified in our study are known as transformational leadership (Lynch et al., 2018; Rutten et al., 2021), which is a dynamic process that focuses on the context, the situation, the self, and others. Transformational leaders are visionary and committed role models (Marshall and Broome, 2017), and they meet all stakeholders whose commitment is necessary for change at an eye-to-eye level (Miles and Asbridge, 2014). A review by Gebreheat et al. (2023) revealed an association between transformational leadership and nurses’ job satisfaction and reported a positive influence of transformational leadership on quality of care, patient outcomes, and of nurses’ intentions to not leave the profession.

In comparison, leadership in practice development includes a bottom-up approach in which stakeholders initiate change and participate in the implementation process (Boggatz, 2022; Manley et al., 2013). This contrasts with our results, which indicate that managers play a central role in influencing or initiating the implementation of the elements of person-centred care while all employees are integrated into the development of a mindset and internal regulations. Therefore, in the context of the implementation of person-centred care for people living with dementia, we agree with Boggatz (2022), who advocates a leadership approach located between top-down and bottom-up approaches.

Our results show that mindset is understood as a central aspect of person-centred care and that its development includes influencing factors, collective understanding processes and mindset work. With respect to factors influencing the mindset of people, the survey results of the Worlds Alzheimer Report 2024 identified an enormous knowledge gap related to the belief that dementia is normal in ageing (Alzheimer's Disease International, 2024). Kong et al. (2022) found that a negative mindset of nursing home staff is a barrier to the implementation of person-centred dementia care in nursing homes, which leads to task-oriented care. The exemplary implementation of the national expert standard of "Fostering and sustaining relationships in care for people with dementia" highlights the relevance of mindset development as an ongoing process (Stehling and Büscher, 2020). Although person-centred care interventions such as case conferences can also be carried out by caregivers without a person-centred mindset, a person-centred mindset will support the sustainable implementation of person-centred care (Stehling and Büscher, 2020).

Our results in relation to mindset development and collective understanding processes are consistent with elements of the international concept of practice development, which is defined as the ongoing development of a person-centred culture (McCormack et al., 2013). The relevance of the collective understanding process in the context of person-centred care is also highlighted in scientific discourse. In Germany, there is no uniform understanding of practice development (Palm, 2022). Nonetheless, the results of this study provide insight into how experts in person-centred dementia care understand the development of a person-centred culture. With respect to the special care of people living with dementia in nursing homes, the experts described a different understanding of normality and a deeper understanding of different forms of dementia.

Person-centred care in nursing homes is the aim of cultural change in long-term care (Jones, 2011; Koren, 2010). Nevertheless, the results of this study show that the implementation of person-centred care can be negatively influenced by external structural requirements, such as those of regulatory authorities. In this context, Byrne et al. (2020) speaks of a system-centred approach to care in which the care provided corresponds less to the needs of the person and more to the needs of the system and the service providers (Kogan et al., 2016). In our study, the participants demonstrated that external quality requirements and audits can hinder the implementation of person-centred care. As a result, leadership in nursing homes creates standardized conventional regulations in a top-down manner that meet external structural requirements but undermine the autonomy of caregivers. This is consistent with research that suggests that the standardization of eldercare through audits does not support staff in developing professional autonomy (Moberg et al., 2018; Pot et al., 2024). Nevertheless, the type of regularization depends on the object (Öhlén et al., 2017). Consequently, a balance is needed between protection against risks, flexibility and consideration of needs and wishes (Carr and Biggs, 2020; Pot et al., 2024).

Regarding internal regulations for person-centred care, our results show that 1) these regulations need to be generated within a continuous process in which a person-centred culture is developed; 2) they need to be generated in a bottom-up manner that includes the care team; 3) they need to be communicated in reflexive processes within the nursing home; and 4) they need to include aspects of mindset development to offer a toolbox for staff to still act autonomously. In relation to person-centred care, Pot (2022) called this reflexive regulation. In addition to self-reflection, reflexive regulation involves aspects such as openness, creativity and cooperation between all stakeholders and the inclusion of the residents’ perspectives as well as the perspectives of their relatives and relevant others (Pot, 2022). On the basis of our results, we suggest that in care practice, internal regulations on person-centred dementia care in nursing homes should not be equated with conventional regulations such as hygiene standards. A redefinition of regulations on person-centred dementia care and best-practice examples is needed to initiate the establishment of these regulations nationwide.

To overcome the situation in which external structural requirements by regular authorities dominate care, our results highlight the need to develop quality criteria that promote the autonomy of care. Pot (2022) suggested that during audits in nursing homes, regulatory authorities should emphasize checking whether staff focus on resident as a person, and consider their needs and wishes. For person-centred dementia care, regulatory work must be realigned so that regulatory authorities are no longer the assessors but are stakeholders in practice who assess each other (Pot, 2022).

With respect to the development of internal regulations for person-centred care, our study highlights the need for nursing homes to have a continuously communicated common understanding that is fundamental to internal regulations. To translate the approach of person-centred dementia care into practice and to develop a common understanding, nursing homes need support and facilitation in the collective understanding process. Since this process of collective understanding is very individual, internal regulations, which are lived by employees, also differ from facility to facility. Accordingly, it makes little sense to consult a checklist (Pot, 2022) or conduct a standardized assessment to measure the existence of internal regulations for person-centredness. Instead, it makes sense to examine the constitution of mindset development towards the joint development of processes and reflexive regulations in the institution. To implement these aspects for quality assessment in nursing in the future, a close discourse with the inspection authorities is required.

### 5.1 Limitations

We planned to include five participants per virtual focus group discussion. However, we were not always able to include five participants per focus group discussion due to coordination conflicts and short-term cancellations. Tuttas (2015) addressed similar unavoidable challenges and recommended a larger group size per focus group to overcome a high attrition rate of 50 %. Nevertheless, in our study, neither participant interaction nor data quality was affected by small group sizes. Instead, we saw the small group size as a benefit that provided participants with space to discuss and share. Furthermore, we conducted only one focus group discussion with scientific experts from Switzerland and Austria. Due to limited time resources, we recruited and included participants from subgroups of practical experts and regulatory authority experts solely from Germany. The results therefore do not allow for a comparison of the perspectives of scientific experts from Switzerland and Austria and those from Germany. However, we expect that the perspectives of nursing practitioners and regulatory authorities differ across countries.

## 6 Conclusions

This study provides insights into person-centred dementia care in German-speaking nursing homes and the differences between internal regulations for person-centred dementia care and regular internal regulations. Our results show how this approach is understood in and for German-speaking nursing homes. The results provide a realistic description of leadership as a hinge function. Leadership is important for the development of a person-centred culture and for the development of internal regulations for person-centred dementia care. This is a continuous process to develop a common understanding of a person-centred culture within a nursing home. We conclude that the development of internal regulations for person-centred dementia care requires both top-down and bottom-up processes: On the one hand, leadership is needed that, among other things, empowers and initiates the collective understanding process and mindset work. On the other hand, assistants, care professionals, quality management officers and managers are required to generate internal regulations about person-centred care. Regulations can be seen as a documentation of their common mindset through a circular, dialogical collective understanding process. Therefore, not only the collective understanding process and mindset work but also the development and application of internal regulations on person-centred dementia care are never-ending, mutually dependent cycles and must be continuously reevaluated and adjusted. The implementation of person-centred care in nursing homes requires freedom from auditing authorities to allow staff to shape residents’ care autonomously.

## List of abbreviations

None.

## Use of data in previous publications

None.

## Availability of data and materials

Access to the qualitative data is possible upon request to the German Center for Neurodegenerative Diseases (DZNE), site Witten. Please contact the data management of the German Center for Neurodegenerative Diseases (DZNE), site Witten (data-management-witten(at)dzne.de).

## Funding

The German Center of Neurodegenerative Diseases e.V. (DZNE) e.V. (grant number N/A) funded this study. The funders were not involved in the research process or manuscript writing.

## Declaration of generative AI and AI-assisted technologies in the writing process

During the preparation of this work, the authors used a deepl translator to improve the readability and language of the manuscript. After using this tool/service, the author(s) reviewed and edited the content as needed and take full responsibility for the content of the published article.

## CRediT authorship contribution statement

**Anna Louisa Hoffmann-Hoffrichter:** Writing – review & editing, Writing – original draft, Visualization, Validation, Supervision, Project administration, Methodology, Investigation, Formal analysis, Data curation, Conceptualization. **Andreas Hohmann:** Writing – review & editing, Validation, Formal analysis. **Bernhard Holle:** Writing – review & editing, Validation, Conceptualization. **Rebecca Palm:** Writing – review & editing, Validation, Supervision. **Martina Roes:** Writing – review & editing, Visualization, Validation, Supervision, Conceptualization.

## Declaration of competing interest

The authors declare that they have no known competing financial interests or personal relationships that could have appeared to influence the work reported in this paper.

## Appendix Supplementary materials

Image, application 1
