# Supplementary material for: Person-centred care of people living with dementia and its regulation in German-speaking nursing homes: A qualitative focus group study
Source: Int J Nurs Stud Adv. 2025 Aug 5;9:100400. doi: 10.1016/j.ijnsa.2025.100400 (PMC12365120; doi:10.1016/j.ijnsa.2025.100400)
Supplement: Supplementary file 1 [file mmc1.docx]

**Appendix**

**Appendix S1**: Consolidated criteria for reporting qualitative studies (COREQ): 32-item checklist

| **No Item** | **Guide questions/description** | **Page No.** |
| --- | --- | --- |
| **Domain 1: Research team and reflexivity** |  |  |
| Personal Characteristics |  |  |
| 1. Interviewer/facilitator | Which author/s conducted the interview or focus group? | 8-9 |
| 1. Credentials | What were the researcher’s credentials? *E.g. PhD, MD* | 8 |
| 1. Occupation | What was their occupation at the time of the study? | 8 |
| 1. Gender | Was the researcher male or female? | 8 |
| 1. Experience and training | What experience or training did the researcher have? | 8 |
| Relationship with participants |  |  |
| 1. Relationship established | Was a relationship established prior to study commencement? | 7 |
| 1. Participant knowledge of the interviewer | What did the participants know about the researcher? *e.g. personal goals, reasons for doing the*  *research* | 7 |
| 1. Interviewer characteristics | What characteristics were reported about the interviewer/facilitator? *e.g. Bias, assumptions,*  *reasons and interests in the research topic* | 8 |
| **Domain 2: study design** |  |  |
| Theoretical framework |  |  |
| 1. Methodological orientation and Theory | What methodological orientation was stated to underpin the study? *e.g. grounded theory,*  *discourse analysis, ethnography, phenomenology, content analysis* | 5-6 |
| Participant selection |  |  |
| 1. Sampling | How were participants selected? *e.g. purposive, convenience, consecutive, snowball* | 6 |
| 1. Method of approach | How were participants approached? *e.g. face-to-face, telephone, mail, email* | 7 |
| 1. Sample size | How many participants were in the study? | 11 |
| 1. Non-participation | How many people refused to participate or dropped out? Reasons? | 7-8 |
| Setting |  |  |
| 1. Setting of data collection | Where was the data collected? *e.g. home, clinic, workplace* | 7-8 |
| 1. Presence of non-participants | Was anyone else present besides the participants and researchers? | 9 |
| 1. Description of sample | What are the important characteristics of the sample? *e.g. demographic data, date* | 11, Table 1 |
| Data collection |  |  |
| 1. Interview guide | Were questions, prompts, guides provided by the authors? Was it pilot tested? | 8-9 |
| 1. Repeat interviews | Were repeat interviews carried out? If yes, how many? | N/A |
| 1. Audio/visual recording | Did the research use audio or visual recording to collect the data? | 9 |
| 1. Field notes | Were field notes made during and/or after the interview or focus group? | 9 |
| 1. Duration | What was the duration of the interviews or focus group? | 8-9 |
| 1. Data saturation | Was data saturation discussed? | 7-8 |
| 1. Transcripts returned | Were transcripts returned to participants for comment and/or correction? | N/A |
| **Domain 3: analysis and findings** |  |  |
| Data analysis |  |  |
| 1. Number of data coders | How many data coders coded the data? | 9-10 |
| 1. Description of the coding tree | Did authors provide a description of the coding tree? | Appendix S4, 11-20 |
| 1. Derivation of themes | Were themes identified in advance or derived from the data? | 9-10 |
| 1. Software | What software, if applicable, was used to manage the data? | 9 |
| 1. Participant checking | Did participants provide feedback on the findings? | N/A |
| Reporting |  |  |
| 1. Quotations presented | Were participant quotations presented to illustrate the themes/findings? Was each  quotation identified? *e.g. participant number* | 11-20, Appendix S4 |
| 1. Data and findings consistent | Was there consistency between the data presented and the findings? | 11-20, Appendix S4, Figure 1 |
| 1. Clarity of major themes | Were major themes clearly presented in the findings? | 11-20, Appendix S4, Figure 1 |
| 1. Clarity of minor themes | Is there a description of diverse cases or discussion of minor themes? | 11-20, Appendix S4 |

**Appendix S2:** Online questionnaire via LimeSurvey

**Questionnaire on internal regulations for person-centred care for people living with dementia in nursing homes**

| *For experts in regulatory authorities* | 1. What comes to mind spontaneously when you think about person-centred care for people living with dementia in your facility? Please outline three aspects in bullet points. [Free text answer] 2. What do you understand the internal regulations of a nursing home to mean? [Free text answer] 3. What do internal regulations for person-centred care for people living with dementia look like in nursing homes? [Free text answer] 4. Which internal regulations in nursing homes include aspects of person-centred care for people living with dementia? [Free text answer] 5. Which aspects of person-centred care for people living with dementia are addressed in the internal regulations of nursing homes? [Free text answer] 6. Which aspects of person-centred care for people living with dementia in nursing homes should be addressed in internal regulations? [Free text answer] 7. Which aspects of person-centred care in specialized dementia care in nursing homes should be addressed in internal regulations? [Free text answer] |
| --- | --- |
| *For science experts* | 1. What do you understand the internal regulations of a nursing home to mean? [Free text answer] 2. What should internal regulations for person-centred care for people living with dementia in nursing homes look like? [Free text answer] 3. What internal regulations should nursing homes have for person-centred care for people living with dementia to enable its long-term implementation? [Free text answer] 4. What internal regulations should there be in nursing homes for person-centred care for people living with dementia in any case? [Free text answer] 5. What internal regulations should there be for person-centred care in specialized dementia care in nursing home in any case? [Free text answer] |
| *For practice experts* | 1. What do you understand the internal regulations in your facility to mean? [Free text answer] 2. What forms of internal regulations regarding person-centred care for people living with dementia exist in your facility? [Free text answer] 3. How are internal regulations developed in your facility? [Free text answer] 4. Do internal regulations for person-centred dementia care differ between traditional care units and dementia-specific care units? If so, why? [Free text answer] 5. What internal regulations should there be for person-centred care in specialized dementia care in nursing homes in any case? [Free text answer] |

**Questionnaire for the collection of sociobiographical and professional biographical information (for all experts)**

| **What is your gender?** |
| --- |
| ☐ male |
| ☐ female |
| ☐ no answer |
| **How old are you?** |
| _________ years |
| **What is your job title?** |
| ________________________________ (free text) |
| **How long have you been working in this profession?** |
| _____________ (years) ______________ (months) |
| **How long have you been working in this facility?** |
| _____________ (years) ______________ (months) |
| **What is your professional qualification? (Multiple answers possible)** |
| ☐ Occupational training as ____________ (free text) |
| ☐ Completed bachelor’s degree: ___________ (free text) |
| ☐ Completed master’s degree: _____________ (free text) |
| ☐ Completed diploma degree: ______________ (free text) |
| ☐ PhD in _____________________ (free text) |
| ☐ Other: _______________________ (free text) |
| ☐ Further training in ______________________________ (free text) |
| **What is your role in your current profession?** |
| ____________ (free text) |

**Appendix S3:** Interview guide

| **Opening question:** | | |
| --- | --- | --- |
| Topic: Personal meaning of person-centred care for people living with dementia | *For science experts, practice experts, experts on regulatory authorities* | What does person-centred care for people living with dementia mean to you personally?   - Please name three terms that you associate with it. |
| **Main questions** | | |
| Topic: Meaning of person-centred care for people living with dementia in nursing homes | *For science experts* | What meaning should person-centred care for people living with dementia have in an organization?   - Please write down in bullet points various aspects of person-centred care for people living with dementia that should be relevant in a nursing home. Please list your bullet points so that you name the most important aspects first. After five minutes, we will present these to the group and discuss their significance. - What do the aspects you listed mean for an organization? - How should person-centred care for people living with dementia in an organization differ from person-centred care that is not focused on one group of people? |
|  | *For practice experts* | What does person-centred care for people living with dementia mean for your company?   - Please write down in bullet points various aspects of person-centred care for people living with dementia that should be relevant in a nursing home. Please list your bullet points so that you name the most important aspects first. After five minutes, we will present these to the group and discuss their significance. - What do the aspects you listed mean for your facility? - How should person-centred care for people living with dementia in an organization differ from person-centred care that is not focused on one group of people? |
| Topic: Meaning of person-centred care for people living with dementia in regulatory authorities | *For regulatory authorities* | What does person-centred care for people living with dementia mean to you as an employee of your regulatory authority?   - Please write down in bullet points various aspects of person-centred care for people living with dementia that should be relevant in a nursing home. Please list your bullet points so that you name the most important aspects first. After five minutes, we will present these to the group and discuss their significance. - What do the aspects you listed mean for the facilities you audit? - What is the difference between person-centred care for people living with dementia and person-centred care that is not focused on one group of people for the facilities you audit? |
| Topic: Meaning of person-centred care for people living with dementia in nursing homes | *For regulatory authorities* | What does person-centred care for people living with dementia mean for the organizations/homes you audit? |
| Topic: Internal Regulations about person-centred care for people living with dementia | *For science experts* | - What makes a (good) internal regulation for you? - What internal regulations should be in place in nursing homes regarding person-centredness for people living with dementia so that a long-term implementation of person-centredness is possible? - Which aspects of person-centred care for people living with dementia are important to you in internal regulations in a care facility? - Optional: To what extent should internal regulations be mandatory for staff? - Optional: Should these internal regulations be documents that entail consequences if these regulations are not complied with? |
|  | *For practice experts* | - How are the aspects you mentioned regulated in your facility? - What do you consider (good) internal regulations in your facility? - Which aspects of person-centred care for people living with dementia are addressed in internal regulations in your institution? - Optional: To what extent are these internal regulations binding for your staff? - To what extent are these internal regulations documents that entail consequences if these regulations are not complied with? |
|  | *For regulatory authorities* | - What do you consider normative points of reference (= guiding, decisive points of orientation) for person-centred care for people living with dementia in a nursing home? - What makes a (good) internal regulation in nursing homes? - How are the aspects of person-centred care for people living with dementia that you mentioned addressed in internal regulations in nursing homes that you have audited thus far? |
| Closing questions | | |
| *For science experts, practice experts, experts on regulatory authorities* | | 1. Is there anything else that you have not yet mentioned but is important to you? 2. Please summarize what you consider particularly important in this discussion. |

**Appendix S4**: Categories and Subcategories and corresponding interview quote

| **Main categories** | **Categories** | **Sub-Categories** | **Quotes** |
| --- | --- | --- | --- |
| **Leadership − the hinge of person-centred care** | Management levels | Top management level | “On the one hand, the whole thing has to be led and initiated by top management.” (Focus-Group-2-Expert03) |
|  |  | Middle management level | “[...] the manager we meet in the facility is usually on my side.” (Focus-Group-9-Expert03) |
|  |  | Lower management level | “[...] because then a leader changes, then care unit manager changes, a very important position in residential care from my point of view [...]” (Focus-Group-2-Expert07) |
|  | Leadership qualities | Dedication | “[...] Above all, you need a very committed manager who also follows up on this, that drives it forwards and supports it to a certain extent. For me, that's almost the most important thing when it comes to implementation. Because in my experience, not only in nursing science but also in practice, is that the fish rots from the head. If it doesn't work, it's often because the management doesn't follow it up.” (Focus-Group-1-Expert00) |
|  |  | Person-centred employee management | “[...] I always engage in relationships and it always depends on what kind of mindset I have, how I interact with them, how the employees interact with each other, I'm always a role model.” (FK PP-001, S03) |
|  |  | Continuity of the leader | “Unfortunately, the reality is that facility managers change more frequently, so all of this has a message, you could say. And that's why I think we need to make it clear that this is a long process that can vary greatly depending on the provider or facility.” (Focus-Group-4-Expert02) |
|  |  | Leadership skills | “When I look at my teams, where it really works, I think that's something that sets the team leaders apart from the other team leaders that they really have this in-depth understanding of what happens, where and why.” (Focus-Group-3-Expert05) |
|  | Leadership consequences | Maintain relationship with employees | “[...] that the providers set themselves up conceptually in such a qualitative way that they can ultimately create a relationship with the employees and thus also lay a foundation to get in better and in a different quality, and thus the spiral goes upwards, as it were, because the fact that the quality on site is oriented upwards means that employee satisfaction is greater […].” (Focus-Group09-Expert01) |
|  |  | Staff continuity | “On the one hand, I think it's really down to this atmosphere, the leaders and the many changes that happen. Because once you've committed to something, even once you've committed to someone at management level and below and said, okay, we're doing this, we think it's good and we're going to implement it this way, then it's not enough, because then a leader changes, then the head of a residential unit changes − a very important position in inpatient care in my view, which has a lot of influence on the team and on how it's implemented, how people are treated. Exactly. And that's where it falls short. And then it somehow gets lost. Then the people who carry it, so to speak, and don't always officially carry it as coaches, but also unofficially exemplify it, they somehow disappear, are lost. Then the person who has always moderated these case conferences is suddenly gone and the whole concept, the whole, here we are with the concept, the whole model, the whole, yes, it all falls apart.” (Focus-Group-2-Expert07) |
| **Setting priorities – ranging from embedding to decoupling person-centred care in a nursing home** | Prioritization of person-centredness | Person-centred requirements | “And the large organizations that operate nationwide are much, much more problematic for us than small facilities, which have completely different requirements in terms of individuality, professionalism and dealing with people or residents.” (Focus-Grouß-9-Expert03) |
|  |  | Prioritization of person-centred structures | “Yes, I would like to bring in another paradox. So I know facilities that have undergone further training, that approach the whole thing relatively innocently, have little idea and provide great person-centred care. And I know facilities that have everything on board, a great environment, great procedures, great quality assurance, and they're the bottom of the pile.” (Focus-Group-2-Expert00) |
|  | Lacking prioritization of person-centredness | Upper management orientation | “And what I also notice very strongly is that the management that we meet in the facility is usually a nursing professional on my side who sees the whole thing in exactly the same way as I do. However, the director of the facility often sees the whole thing differently. And then we have a problem because the management is not free to make their own decisions.” (Focus-Group-9-Expert03) |
|  |  | Profit orientation | “If I am a company that only has one goal, to pay out a dividend that is as high as possible, then I would say that I am not interested in person-centred care at all. Nobody audits it in this form because it cannot be audited in this way. And why should I invest anything in it? It only costs money.” (Focus-Group-8-Expert03) |
|  |  | Regular authority orientation | “It culminated in the sentence that my NHM [nursing home manager] said to me: if I have to decide between the requirements of the MRB [medical review board] and those of the residents, I will always choose the MRB. That, THAT is something that I think we often encounter, that there is often such a fear-driven attitude, especially at the level of those responsible, that I am doing something wrong externally and that prevents those responsible from actually focusing on what the job is.” (Focus-Group-8-Expert01) |
| **Mindset – ranging from development to stagnation** | Influencing factors | Society’s recognition | “And socially, there is often no acceptance at all. Here, too, it is often the case that working with people living with dementia is not recognized by society at all. Once again, I'm talking about people living with dementia. I know I have a tendency to do that (smiles), but they are often highly dependent and are often not heard at all or are not heard by society.” (Focus-Group-9-Expert01) |
|  |  | Generation of employees | “But it was also a process, of course. Now it's also a process again, because I'm noticing a generation change in our facility, and not just among the employees […].” (Focus-Group-6-Expert01) |
|  |  | Expertise of employees | “[…] the knowledge must be there. The knowledge of the expert standard must perhaps also be there. Then the staff who implement it must be appropriate. They must also be sufficiently trained […].” (Focus-Group-8-Expert04) |
|  |  | Individual mindset of employee | “So without an understanding or a personal mindset, well, you always have that. Well, you always have it, so you always have some kind of understanding or some kind of mindset. However, if it runs contrary to the considerations of person-centred care and I come in with the mindset, so actually it's only about being full and clean in an exaggerated sense, now let's stick with that. Nobody will say that nowadays, but then it wouldn't be possible. So then it's already a basic condition [...]” (Focus-Group-4-Expert02) |
|  | Collective understanding process | Inclusion of all perspectives | “I believe that if the team and the management, i.e. all those who are on site, therapists etc., also engage with these issues, including person-centeredness, then a positive attitude can be generated.” (Focus-Group-9-Expert01) |
|  |  | Communication | “I would also understand person-centeredness not only…, if we now look at the, the field …, I'll say the elderly care sector or facilities, I would not only focus on the residents, but also on communication in the entire facility. I would understand this to include communication among the employees, communication from the top management to the base, as well as the other way around, as well as considering the residents and relatives. Therefore, the overall topic of communication, how does this take place? Are other ideas and perspectives also included? To what extent does this in turn influence the work with the residents on site [...]?” (Focus-Group-9-Expert01) |
|  |  | Shared dynamics | “And when a team has to find itself and, under certain circumstances, more and more people with very different migration biographies and a very different understanding of care come together, you have to somehow try to commit to a completely dynamic, continuous process that must never stop. But we have to commit to or agree on something, because otherwise we're swimming far too much.” (Focus-Group-2-Expert03) |
|  | Mindset work | Designing environment | “Yes, and environmental design, especially with regard to the inner milieu, it's really about these points, such as self-determination, recognition and appreciation, respect, so that it is possible to create such an environment in the care units.” (FK NP 02, S00) |
|  |  | Understanding the resident with dementia as a person | “Personal freedom, that I can be who I want to be, that I have space, or that I have room for it and that I can shape my life the way I am and the way I want to.” (Focus-Group-1-Expert01) |
|  |  | Individualizing care | “Recognizing individuality. Yes, for me it's also very important… it's also a fundamental in any case, that you don't lump everyone together, but really look at the individual needs and precisely what can't be expressed or can't be expressed verbally. But perhaps it's also difficult to recognize otherwise, that special consideration is given to this, no, biography is of course always taken into account, but you also look at what has perhaps been done a lot biographically and perhaps no longer wants to be done because of this.” (Focus-Group-5-Expert04) |
|  |  | Changing perspective | “The mindset means that when I provide person-centred care, it is, yes, in palliative care one would say a radical orientation towards the person affected. In other words, I don't see my needs as a caregiver, but I try to see this position, the other person's point of view, and to orient myself towards it as much as possible. And that means accepting the other person, which is also an attitude, which is why it is an appreciation, which is why it fits in with the competence of validation. But basically it's not a technique, it's how I treat the other person. And in doing so, I incorporate their wishes and memories; that's part of it for me. So if I take this position, accept the other person, take myself back, in other words this interaction, which would be balanced somewhere, let's say, between equally competent people I have to take the side of the person concerned, the person affected, and do what they don't do in communication in order to maintain their personhood in this communication.” (Focus-Group-1-Expert00) |
|  |  | Providing activity supplies | “Then, I also had the idea that the offers for shaping relationships should be as individualized as possible, which means that not everyone has to take part in any group activities (smiles), but that you really look at what this person needs.” (Focus-Group-2-Expert06) |
|  |  | Designing relationship | “Then, […], for me, this aspect of initiating a relationship is really something very essential, important, that I, do it really well… I really think about it for each resident, discuss it in the team, even if someone has found access, so to speak, that it doesn't remain his secret, so to speak.” (Focus-Group-5-Expert-04) |
|  | Preliminary consequences of collective mindset development | A shared understanding | “Therefore, for me, something like an understanding is something that actually has to be established again and again every day through certain process specifications.” (Focus-Group-2-Expert00) |
|  |  | Culture development | “Yes, but perhaps first of all to create a culture like that. Well, we had just said, okay, we have to involve the employees anyway. I mean, we also have changes of employees. However, in order to develop a basic position, a basic attitude somehow, it would perhaps be quite good to really capture all perspectives. And to develop this together, yes, to develop a basic culture, a person-centred culture in the institution.” (Focus-Group-1-Expert02) |
| **Structural requirements – range from self-determination to independency** | External structural requirements | Regular authority requirements | “And I think what then also needs to play a role, and this is the big challenge for me at the moment, is that we see that we once again have a high conceptual standard. However, we also know that everyday life is triggered by the question of what is obligatory in terms of quality. And then, of course, we have a gap, and if this is not reflected in the quality assurance that is carried out for the external obligations, which I actually consider very important, then of course I run the risk of becoming reductionist again, because I naturally say that the main thing is that I meet the quality criteria so that my facility does well here. And I think that the management or leadership of such an institution has a special responsibility because they have to make it clear that one thing is the quality discourse, which is mandatory and defined externally, and the other is our basic understanding or our basic attitude.” (Focus-Group-4-Expert01) |
|  | Institutional structure | Facility structure | “What does it actually look like in our institution and what do we perhaps need to make more flexible or different? Exactly, I believe that an institution that has very rigid structures can also have difficulty implementing person-centeredness.” (Focus-Group-2-Expert05) |
|  |  | Personnel structure | “Well, you would like to, but you can't, for example due to a lack of staff because a sick call came in at short notice. And that's a shame, of course, but as has already been said, you have to look after them. Unfortunately, you can't do it as individually as you would like and as it would be good for the residents.” (Focus-Group-7-Expert03) |
|  |  | Care structure | “Yes, the size of the living group should not exceed twelve people. Therefore, no areas with 30 or 40 people. These are basically just storage facilities.” (Focus-Group-2-Expert00) |
| **Internal regulations – range from microstandards to a person-centred toolbox** | Types of regulations | Mission statements | “So that can start with the mission statement, where all the things are already described. In day-to-day interaction and care, when it comes to the topic of dying, this is also part of the external image that you give. Quite apart from the fact that there are also requirements that have to be fulfilled, you also have to present your institution.” (Focus-Group-7-Expert03) |
|  |  | Quality manuals | “[…] everything relating to nursing care is written in the quality manual.” (Focus-Group-4-Expert-01) |
|  |  | Operationalized written procedures and structures | “[…] perhaps even more, a clear procedure, especially when it comes to particularly important cases.” (Focus-Group-1-Expert00) |
|  |  | Documentation | “Documentation is always a regulation, isn't it? So it's also important to record things so that they don't get lost and disappear.” (Focus-Group-7-Expert01) |
|  | Relevance of regulations | Professionalization of nursing | “However, at the end of the day, there are still things that are supposed to set the tone in certain areas. That is also important. We have all said that by consensus. So that care also has a certain professionalism.” (Focus-Group-6-Expert00) |
|  |  | Presentation of external structure requirements | “So at least it has to be stated somewhere that you work like this. You have to file it, also for the MRB [Medical Review Board], for the home supervisory authority, whatever.” (Focus-Group-7-Expert01) |
|  |  | Process security | “I think it is important that there is process security in institutions, and perhaps I think this because I am relatively far away from you. This means that there needs to be clarity in institutions, that it should be regulated who is in what role when, who has what task when?” (Focus-Group-1-Expert-01) |
|  | Characteristics of good regulations | Updated | “I really try to keep it manageable when I write concepts for myself so that I can look at them after a while and revise them and don't need three days every time.” (Focus-Group-6-Expert00) |
|  |  | Digitalized | “Thank goodness it's now digitalized, so that you don't have 20 folders lying around somewhere, but can actually read through it piece by piece on your PC.” (Focus-Group-7-Expert01) |
|  |  | Compactness | “I think it should be short and concise, because nobody reads through 20,000 pages.” (Focus-Group-6-Expert02) |
|  |  | Practicability | “[…] that it is manageable and practicable, I would say.” (Focus-Group-1-Expert02) |
|  |  | Comprehensibility | “So that it is also understood by practitioners.“ (Focus-Group-1-Expert00) |
|  |  | Target-group specific | “Target group-oriented. So that actually picks up on what I just said. I don't think there can be a single regulation or set of regulations; they have to be differentiated in such a way that they suit different audiences.” (Focus-Group-4-Expert02) |
|  |  | No standardization | “Yes, maybe, but what definitely won't work is a recipe book.” (Focus-Group-2-Expert00) |
|  | Regulations on person-centred dementia care | Development of regulations on PCC | “Yes, perhaps an essential element from my point of view is that employees are involved in the development of these instruments, however you want to define it now, the regulations. That they are not necessarily prescribed top-down, but that we set out together, based on whatever kind of initial understanding of good, or good care for people living with dementia, perhaps person-centred care for people living with dementia, and then work together to develop them.” (Focus-Group-1-Expert-04) |
|  |  | Communication of regulations on PCC | “And I think that the management or leaders of such an institution naturally have a special responsibility because they have to make it clear that one thing is the quality discourse, which is mandatory and defined externally, and the other is our basic understanding or our basic mindset. And it is at least as important to me that these requirements continue to be met. And I could well imagine that, if you put it across, because everything that concerns nursing care is written in the quality manual, it would be cross-checked, so to speak.” (Focus-Group-4-Expert01) |
|  |  | Content of regulations on PCC | “For me, indicators would be described as follows: how do we create a situation of equality here? So how, with what measures (...) do we try to ensure this? One example could be that a care plan is always evaluated in the presence of the resident and not in the evening when I have just an hour to spare, but in conversation with the resident. In the best case scenario, the resident gets to read it and has to confirm that what I have written is correct and corresponds to his or her view.” (Focus-Group-9-Expert03) |
|  |  | Understanding of regulations on PCC | “[...] where relatives in particular but also other visitors, should be made aware that when they enter the protected areas with people living with dementia, they are now leaving the normal world, so to say, and entering the protected area. And there are various sentences and rules that you ask the visitors for. It's essentially about all the things we've discussed: self-determination, well-being, being able to let go, your own world of experience, being accepted.” (Focus-Group6-Expert00) |
|  |  | Alternative term for regulations on PCC | “And the framing will then be, then I'll close it, then I'll stop, would be this toolbox. So a collection, a conglomerate of procedures that you could use if you say, I want an input, oh, just look to see if there's anything in our standard folder that you can use. Although the word standard almost bothers me. Instrument case, possibilities, how can you interact or what can you do? That's my idea off the top of my head.” (Focus-Group-8-Expert03) |
| **Outcomes fro person-centred development** | Outcomes at facility level | Atmosphere | “[…] a culture of closeness and warmth.” (Focus-Group-6-Expert00) |
|  |  | Staff confidence | “So this, I have the knowledge, I have the experience, I have the evidence, I can do this and I am self-confident and can also represent this to other people. Period.” (Focus-Group-2-Expert03) |
|  |  | Professionalism | “And professionalism, because I simply believe that this is truly professional care, when we have the ability to respond individually to this situation, to act, this reflective practice in the situations. I simply see how much it takes.” (Focus-Group-3-Expert05) |
|  | Outcomes at individual level | Self-efficacy | “And then I think a central aspect is that self-efficacy must be tangible for everyone involved. For the resident, of course, this means that they at least feel their self-efficacy in a positive sense and therefore don't necessarily have to shout and scream and the like. However, above all for the staff.” (Focus-Group-3-Expert04) |
|  |  | Positive influence on resident | “And the goal, the whole person-centred care aims to ensure that someone has a good life. A good life from their point of view. What the person understands by that is up to them. They have to find out to a certain extent. It's very different. It's individual, but that's what it's all about.” (Focus-Group-1-Expert00) |

**Appendix S5** Detailed description of perceived leadership qualities

Due to the limited word count in the manuscript, the category “leadership qualities” and its subcategories are summarized in a table.

| **Aspect** | **Description** |
| --- | --- |
| Dedication | - The leader, as an open person,   - requires a clear, person-centred understanding and a concrete goal at management level.   - initiates the ongoing process of collective mindset development.   - scrutinizes the nursing home together with the employees to conceptually reposition the organization.   - not only supports and promotes the person-centred understanding in the institution but also claims it. |
| Person-centred employee management | - The leader   - Requires a state of balance and peace with employees.   - is responsible for organizing and receiving training on person-centredness.   - expresses a person-centred mindset through appreciative interaction with employees, leads by example in person-centred action, and sees employees as the most valuable asset.   - leads the person-centred attitude by example, including 1) responding individually and 2) communicating transparently with employees.   - is undertaking facilitative leadership, in which employees are given sufficient freedom to develop a person-centred mindset that they express through their actions.   - enables facilitation by providing key persons who accompany and promote person-centred mindset development as moderators, contact persons, multipliers and trainers. By supporting and accompanying collective communication efforts in the form of transparent communication, reflection and guidance, facilitation makes it possible to have an influence on the team, navigate the process and find joint solutions.   - is aware that his or her counterparts cannot only anticipate their own mindset but can also adopt this mindset over time. For this reason, leaders see themselves as role models who exemplify a person-centred mindset to employees through their interactions and other attitude work. Taking on a role model function involves training of employees, leading person-centred interaction with residents by example, reflecting on actions together after observation of nurse-resident interactions and discussing possible solutions. |
| Continuity of the leader | - Leader continuity enables   - the continuity of the team and maintaining the internally developed concept of person-centredness. |
| Leadership skills | - Interaction-related skills   - With their ability to observe, leaders see employees from all professions, anticipate their attitudes from their actions and assess whether these match or deviate from their own attitudes.   - This includes the manager selecting staff based on the jointly developed understanding of person-centred care and parting ways with those who do not have the same understanding.   - They also support the implementation of person-centred care towards the managing directors. - Hermeneutic competence   - The leader recognizes what happens in the care unit, for what reason and by which employees it is initiated.   - Knowing the employees as persons. - Empathy   - A skill that provides leaders with the sensitivity to deal with employees on an individual basis. This also means, for example, assigning employees to individual shifts. - Professionalism   - A skill that enables leaders to respond to the individual person and in these situations through reflective practice. - Pedagogical competence of guiding employees   - Creating a learning environment. |

**Appendix S6** Detailed description of the perceived collective understanding process

Due to the limited word count in the manuscript, the category “collective understanding process” and its subcategories are summarized in a table.

| **Aspect** | **Description** |
| --- | --- |
| Inclusion of all perspectives | Inclusion of all perspective means the integration of   - all persons in the nursing home, such as managers, nurses, nursing assistants, the person with dementia, home economics, service engineering. - the people working outside the nursing home including therapists, visitors, and vendors. |
| Communication | - Communication   - as a dynamic, continuous and ongoing process.   - comprises both the dynamics of verbal and nonverbal communication - Communication tools:   - Collaboration with family members, dementia care mapping and supervision, as well as staff meetings, case conferences and shift handovers. - Communication forms:   - A regular dialogue is notable for an occasion-related form of communication of talking to each other with the goal of permanently reflecting and further developing a common understanding of care and a common mindset.   - The negotiation process within the team involves a joint dialogue about personal and shared understanding and the implementation of person-centred care. The aim is to reach a joint consensus or compromise on a common understanding that provides a framework for mindset development and mindset work.   - The constant dialectic within the team includes continuously and reflexively identifying the different perspectives of employees and working with them to achieve a new perspective with the goal of constantly remapping and renewing the shared understanding.   - Reflection is a processual meta-communication resource that employees use both as a communication form and as a communication tool. Its aim is to push a lasting optimization and further develop mindset and culture in the nursing home by asking about understanding, actions, and employees. As a communication form, reflection is used for self-reflection as well as for bilateral and interprofessional reflection within the team. The content includes reflections on person-centredness at facility, staff, and resident interaction level. Reflection also represents meta-communication to reflect previously named communication forms as well as communication tools for processes or procedures. |
| Shared dynamics | - A shared dynamic within the team to maintain a common understanding. This means a reflexive, continuous process that features a common mindset in the team and the resulting appreciative interaction, mutual trust, empathy, open-mindedness, transparency and commitment. The continuous process sets itself apart by various influences, which is why it can be associated with a wave motion. |

**Appendix S7** Detailed description of perceived mindset work

Due to the limited word count in the manuscript, the category “mindset work” and its subcategories are summarized in a table.

| **Aspect** | **Description** |
| --- | --- |
| Designing the environment | - Design of physical environment, which considers and adapts the needs of the residents and staff. - Design of the inner milieu sets itself apart by equality, in which every person is treated equally. - Inner milieu includes principles of dealing with people living with dementia, such as self-determination, recognition and respect.   - Appreciative and reflective communication entailing validating conversations and patience.   - Speaking a language that creates access to the resident by using different terminology or non-everyday language concepts. |
| Understanding the resident with dementia as a person | - The person as a constantly re-established unity of all his or her connections and relationships in the past and in the present. - Being a person means enabling the person living with dementia to live a self-determined life in which they can be who they want to be and in which staff account for their will and decisions. |
| Individualizing care | - Knowing the person with dementia. - Biographical work, which includes experiences, emotions, meanings and socioeconomic status. - Intensive work with the person's information on personal issues, the relevance system, basal needs and preferences. - Staff collect this information and use it for further interaction after validation by the resident. |
| Changing perspective | - Ability to focus on the person’s affectedness. - Attempt to understand the perspective of the person with dementia by withdrawing one’s own presuppositions and forming a hypothesis of understanding. - Perspective includes references to the person, such as his or her behaviour, way of communication and understanding of a good life. |
| Providing activity supplies | - Addresses relationship building, social interaction and working with individual preferences. - Include housekeeping, individual and group activities with various board games. - Dementia-specific interventions are developed and implemented explicitly for people living with dementia and include measures that focus on communication with the person with dementia. |
| Designing relationship | - Includes relationship building, continuity and maintenance of family relationships and other contacts in the social environment as well as inclusion through involvement in the community. - Holistic view of the person. - Entering a relationship requires engagement in the interaction by being present and aware. - Designing relationship also includes positive interactions that strengthen personhood and thus positive feelings and support actions that can no longer be carried out independently. |

**Appendix S8:** Detailed description of perceived structural requirements – institutional structures

Due to the limited number of characters in the manuscript, the category “structural requirements” and its subcategories are summarized in a table.

| **Aspect** | **Description** |
| --- | --- |
| Facility structure | - Framework conditions include   - Changing conditions in the nursing home, such as changes in the resident clientele.   - Hindering conditions regarding the staff, such as the increase in staff shortages, the skill decline of young care professionals and the associated decline in professional care for people living with dementia.   - Fostering conditions in resident-staff-interaction include enabling conditions, which are necessary for acting in a person-centred and supportive manner in the interaction with the resident: flexible structures with sufficient personnel and physical, temporal, social and personal space to engage with the resident individually.   - The environment within a nursing home includes the interior design, which can support person-centeredness or hinder relationships (e.g. in four-bed rooms without space for relatives to sit down). The environment outside the nursing home includes the habitat and social environment for the resident. - Quality management includes developing regulations. - Training opportunities with different training concepts.   - Intrinsic motivation includes supplies for all nursing home employees, e.g. the basic geriatric psychiatry course for all staff involved in care but also the use of multipliers who pass on content to employees as well as practical support and reflection through practical guidance.   - Training supplies for external motivation include regular trainings and gerontopsychiatric specialist training. |
| Personnel structure | - Fostering structures include personnel planning, the skill and grade mix and sufficiently trained personnel. This includes qualified and academic staff, as well as gerontological-psychiatric nurse specialists. - Hindering personnel structures include the low staff to resident ratio, which thrives through staff illness and appears alongside shortcomings in care. |
| Care structure | - A supportive structure   - Includes a flexibly created daily structure, action plan and care plan. Staff use these documents on the one hand to record the individuality of the resident and gain sufficient information and on the other hand to meet the quality criteria of the regulatory authorities.   - Is a care unit concept that differentiates between integrative and segregative concepts. These care unit concepts define the structure of the care unit and staff, the staff presence and the resident clientele as well as the size of the care unit defined by the number of residents. |

**Appendix S9:** Researcher Description

Researcher description

The first author is female and worked as a registered nurse. She has a master’s degree in nursing science and is employed as a research associate. She has been working on the topic of person-centred dementia care and internal regulations in nursing homes for three years. The second author is a male qualitative social researcher. He has a master’s degree in educational science and is employed as a research associate. The second author has not previously worked on this topic of study. Both the first and the second author have experience in qualitative research and bring different levels of prior knowledge to the data analysis based on their backgrounds. The rest of the study team (Author 3, 4, 5) consists of experts with many years of experience in health services and dementia research as well as various qualitative and quantitative methods.

**Appendix S10:** Quality Criteria

This study met the quality criteria for qualitative research according to Steinke (2004). To ensure intersubject comprehensibility, we documented the research process of this study stepwise and transparently. In addition, we discussed the entire research process and the results of the data analysis in monthly meetings with the research team. To adopt the peer debriefing approach, the first author reflected on the project at regular intervals with colleagues from the German Center for Neurodegenerative Diseases (DZNE) who were not involved in this project. For the indications of the research process, we have transparently presented research design and methods. For empirical anchoring, we have underpinned the results of this study with sufficient citations that we translated into English. Furthermore, we have explicitly presented and justified the relevance of the work. In the discussion section, we have specified the need for research derived from the results. The first author wrote memos before and after each focus group discussion as well as during data analysis for reflected subjectivity. The first author used regular meetings with the research team and colleagues for self-reflection.

Steinke, I. (2004). Quality Criteria in Qualitative Research. In U. Flick, E. von Kardoff, & I. Steinke (Eds.), *A Companion to Qualitative Research*. SAGE Publications Ltd.
